# Navigating polycrisis: long-run socio-cultural factors shape response to changing climate

**DOI:** 10.1098/rstb.2022.0402

**Published:** 2023-11-06

**Authors:** Daniel Hoyer, James S. Bennett, Jenny Reddish, Samantha Holder, Robert Howard, Majid Benam, Jill Levine, Francis Ludlow, Gary Feinman, Peter Turchin

**Affiliations:** ^1^ Complexity Science Hub, 1080 Vienna, Austria; ^2^ Evolution Institute, San Antonio, FL 33576, USA; ^3^ University of Washington, Seattle, WA 98195, USA; ^4^ Trinity College Dublin, Dublin, D02 PN40, Ireland; ^5^ Field Museum of Natural History, Chicago, IL 60605, USA

**Keywords:** polycrisis, global history, societal crisis, collective action, cliodynamics, Seshat

## Abstract

Climate variability and natural hazards like floods and earthquakes can act as environmental shocks or socioecological stressors leading to instability and suffering throughout human history. Yet, societies experience a wide range of outcomes when facing such challenges: some suffer from social unrest, civil violence or complete collapse; others prove more resilient and maintain key social functions. We currently lack a clear, generally agreed-upon conceptual framework and evidentiary base to explore what causes these divergent outcomes. Here, we discuss efforts to develop such a framework through the Crisis Database (CrisisDB) programme. We illustrate that the impact of environmental stressors is mediated through extant cultural, political and economic structures that evolve over extended timescales (decades to centuries). These structures can generate high resilience to major shocks, facilitate positive adaptation, or, alternatively, undermine collective action and lead to unrest, violence and even societal collapse. By exposing the ways that different societies have reacted to crises over their lifetime, this framework can help identify the factors and complex social–ecological interactions that either bolster or undermine resilience to contemporary climate shocks.

This article is part of the theme issue ‘Climate change adaptation needs a science of culture’.

## 1. Polycrises of the present and past

Increasing awareness exists among academics, policy analysts, and the public that we are experiencing a global ‘polycrisis': a series of interconnected and interacting threats—climate change and ecological disasters, rising economic inequality and political polarization, violent conflict and more (cf. [1]). While the scale and global synchrony of these threats present novel challenges, these types of stressors have been an inherent part of the human experience, and have at times converged with other socio-cultural pressures; *there have been polycrises in the past as today*.

Analyses of our current polycrisis often overlook lessons offered by historical examples, while scholarship exploring past environmental crises typically converges on a few well-known cases of severe stress contributing to apparent societal collapse [2–8]. These studies generally focus on a single event or particular social formation, seeking to establish temporal correlations between environmental hazards and major societal transformations. While the resulting findings are of intrinsic interest, they typically do not offer insights generalizable to multiple cases from different regions and periods.^1^ Further, while the transformations often associated with collapse are frequently presented as the extreme outcome of ‘failures' to adapt or develop resilience against challenges, such ‘collapses’ may themselves be considered powerful adaptations required to replace maladaptive or otherwise vulnerable systems with more resilient ones. A positive development has been a recent surge in work across and between disciplines—anthropology, environmental and biophysical science, sociology, history, among others—attempting to grapple with the inherent complexity of contemporary and historical human–environmental relations, highlighting the diversity of societal responses to adverse climate and ecological shocks (cf. [12–15]). Such work has identified many open questions, not least about societal and environmental interactions at different temporal and spatial scales: why, for instance, do large-scale climate events tend to produce divergent outcomes among different societies? What are the principal forces underpinning social vulnerability? Also, why do social systems seem to oscillate between sustainability and vulnerability over long time periods?

Here, we describe some key insights from our study of past crises along with other recent work by colleagues as part of the Crisis Database (CrisisDB) research programme. Our survey of the historical record captures a diversity of outcomes following crises, ranging from major civil wars and societal collapse to less disruptive events and even positive outcomes—namely, systemic reform that can promote well-being or increase democratic participation. One key initial finding is that slowly developing structural forces may undermine social resilience by pressuring governance systems and creating fissures between groups within a society, eroding the social cohesion (and hence capacity) required to collectively respond to threats and to maintain stability. In our view, evolving socio-cultural structures can *prime* societies towards particular responses and so mediate the impact of environmental hazards. Whether a society thus exhibits resilience, positive adaptation, or alternatively maladaptation and/or collapse is thus a function of not only the type and severity of stressors experienced; it is equally a matter of the *vulnerability* in underlying socio-cultural structures that develop gradually over time.^2^ Put simply, the societal impact of any environmental stressors is fundamentally a ‘co-production' of ecological and structural forces interacting through space and time [19].

We conclude by highlighting directions for future research into the interaction of environment, political and economic structures, and cultural developments, noting the importance of such work for policy recommendations to help contemporary societies navigate ongoing and future challenges. Ultimately, we argue that a science of climate resilience and adaptation requires a science of cultural evolution.

## 2. Complex relationships between environmental and societal dynamics: problems of scale and scope

**Figure 1.**
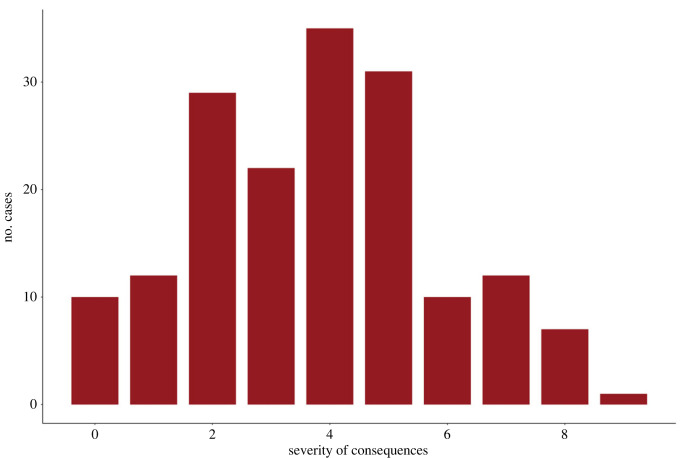
Number of cases in the CrisisDB historical sample experiencing different types of unrest and instability (*n* = 169). Severity of consequences are calculated as a cumulative total of 13 possible outcomes. See the electronic supplementary material for details.

We have systematically collected information about the character and consequences of over 150 past societal crises covering multiple world regions at different historical periods.^3^ Figure 1 illustrates the wide range of outcomes experienced by past societies. These range from more severe cases where multiple destabilizing events overlap (uprisings, civil war, mass mortality, societal collapse or fragmentation) to less disruptive examples wherein ruling classes are targeted (assassinations, elite extermination) or where only a few events co-occur,^4^ to a few cases where none of these happen or are not attested in the available sources.

As noted above, many researchers have viewed environmental degradation, often occurring in tandem with periods of climatic change, as primary drivers of societal collapse. Yet, reviewing a large sample of cases from different places and times reveals an inconsistency: not every ecological shock or climatic anomaly leads to collapse or even severe crisis, while not every crisis involves a major environmental stressor. Some well-known crises in our sample did occur under adverse environmental conditions and led to severe consequences—the collapse of the Sassanid Persian Empire and conquest by Muslim forces in the seventh century CE came in the midst of upheaval, disease outbreak and the climatic stress of the Late Antique Little Ice Age; the Bourbon Dynasty in Medieval France experienced significant civil unrest and social transformation during the so-called Maunder Minimum; the Ming Dynasty in China fell amidst famines, epidemics and unrest in the heart of the Little Ice Age; Aztec and Inca populations were decimated by poor climate, major epidemics and civil unrest even before the Spanish conquest. Other severe crises, however, occurred without major apparent environmental stressors—the fall of the Russian Romanov Dynasty at the turn of the twentieth century or of the Chinese Tang Dynasty in the tenth century, or Rome's transition from Republic to Principate at end of decades of civil war at the turn of the first millennium CE. Conversely, several societies survived serious hazards without major loss of state functioning—the Byzantine and later Ottoman Empires both survived decades of adverse conditions, famines, plagues and other stressors; the Phoenicians in the Levant and New Kingdom Dynasty in Egypt survived the massive climate shift that may have brought an end to a host of societies across Eurasia as part of the so-called ‘Bronze Age collapse’; several Mesoamerica urban centres not only survived but thrived within the same drought conditions that played a role in propelling the Maya collapse. Likewise, favourable conditions are often cited as propelling societal growth, as with early urbanization during a wet period in the fourth millennium BCE Mesopotamia, or the warm periods that helped propel Roman expansion throughout the Mediterranean in the late first millennium CE and fuel the rise of the Killke (precursors of the Inca) in highland Peru. Here too, though, we fail to see any consistent pattern, as not every society impacted by periods of nominally favourable conditions experienced similar growth.

**Figure 2.**
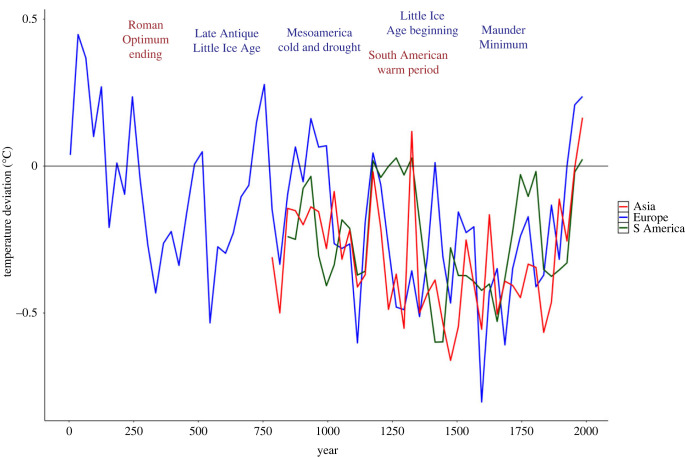
Global anomalous temperatures in °C compared to the 1961–1990 mean, showing dynamics for East Asia (red), Europe (blue) and South America (green). Major climate periods mentioned in text are labelled. Data from the PAGES project [25,26].

An initial conclusion, then, is that while environmental forces may play varied and sometimes critical roles, they can never alone explain observed historical dynamics. To achieve more complete understandings there is, moreover, a need for studies ranging in spatial focus from highly localized to regional and inter-regional levels, in temporal focus from single events to longue durée environmental and societal (e.g. demographic, cultural) developments, and in human focus from individual behaviours to cross-cultural comparisons (e.g. [20,21]). A related challenge is to ensure that the scope of study (including evidence base and methodology) is appropriate for the scale of the environmental forces investigated. Some such forces are inherently macro-scale phenomena impacting large territories that contain multiple societies and may play out over extended periods, from decades to centuries (figure 2). Yet, as noted above, typically studies assessing climatic impacts on social change focus on individual societies within the catchment area of these environmental effects, seeking straightforward correlations between environmental and societal events or relating the magnitude of societal disruption to the level of the environmental distress felt. Only by exploring the reactions of all (or at least many) societies impacted by a particular climate ‘regime' is it possible to determine the causal potency and general efficacy of the environmental stressor. When such a broader scope is employed, different societies are generally observed to have quite divergent reactions. For instance, Peregrine recently explored a wide selection of societies from the Little Ice Age period often implicated as causing major societal disruptions across Eurasia, finding that not every society impacted by this cooling experienced comparable disruption [22]. Likewise, scholars continue to disagree about the role of adverse climate conditions in felling a number of large imperial polities in China over the past two millennia; some argue that the correlation between environment and socio-political or economic crises is inconsistent across cases [23], while others point to a persistent temporal association between volcanically forced cooling and dynastic transition or collapse, at the same time noting that the scale of volcanic forcing operates interactively with the scale of pre-existing socio-political and economic stress [24].

Other stressors, including weather-related phenomena such as floods or droughts and associated ecological shocks, are more regionally and temporally focused than evolving climate regimes and generally better matched to the social scales that are typically explored in historical accounts. Scholars have cited major droughts as the root cause of collapse in societies that include Classic Maya civilizations and other urban centres throughout Mesoamerica, the Akkadian Empire of west Asia, the Angkor Kingdom in Cambodia, the Rapa Nui peoples of Easter Island, the Puebloan peoples of the southwestern USA and a host of other cases.^5^ Such arguments generally hold that prolonged or acute and recurrent drought disrupts food production, triggering famines that promote disease, foment unrest, and can eventually spiral into mass mortality, civil violence, site abandonment, and socio-political breakdown and/or transformation. Compelling correlations undoubtedly exist between ecological stressors and major societal change in some such instances, but less clear is whether these arguments are generalizable across cases. More significantly, lingering questions persist over how precisely these ecological shocks produce the observed societal changes, particularly when viewed from a broader scope that reveals the divergent reactions that even the same society can have to a shock at different times. Indeed, many instances of major drought can be adduced that did *not* lead to terminal disruption or collapse. For instance, polities that faced and overcame significant drought include the Mughal Empire in South Asia, the Ptolemaic Kingdom centred in Hellenistic Egypt, and the Ashikaga Shogunate in medieval Japan, the latter comparable in territory and population size to the polities of the Classic Maya. Perhaps recurrent (additive or compounding) stress from droughts sufficiently weakened certain states' functioning and resilience, contributing to their ultimate downfall (e.g. the British conquest of India; the splintering into a warring-states period in Japan), though exactly how recurrent ecological stress acted upon state vulnerability or why some succeeding regimes better managed these same stressors still requires explanation. Even cases often seen as straightforward examples of climate-led collapse have recently been challenged. Kohler and colleagues, to cite one prominent example, note that while severe and prolonged drought contributed to the abandonment of many Puebloan settlements in the thirteenth century, these societies had been declining from overpopulation, diminishing productivity and destabilization from internal violence before the onset of major ecological stressors [27].

The experience of Early Modern France and England further illustrates these complexities well. This period fell within the anomalously low temperatures experienced during the European Little Ice Age (figure 2), exacerbated by recurrent explosive volcanism and, between roughly 1645 and 1715 CE, by reduced sunspot activity termed the ‘Maunder Minimum' [28] (figure 2). These conditions precipitated major stressors such as failed harvests and famine, and probably contributed to the recurrent epidemics during this period, including smallpox, typhoid, influenza and the reappearance of the bubonic plague. Both England and France faced these same environmental–ecological stressors and both experienced major political turbulence during the 1640s–1650s, which at first blush may suggest a causal relationship.^6^ Yet, the unrest was not confined to the period when environmental conditions were poorest and, apart from the mid-seventeenth century crises, major civil wars in England and France did not coincide in time.^7^ Between 1789 and 1870, when climate was relatively improved, France experienced a series of revolutions. Despite sharing the same broad environmental context during this period, England avoided revolution. Although popular immiseration, particularly among industrial labourers, fomented unrest in England during the first half of ninteenth century, structural pressures remained relatively low and the state maintained considerable capacity, leading to much less disruptive unrest and institutional reform compared to France.^8^

## 3. Structural factors mediate environmental impacts and shape societal responses

If societal response to environmental stressors is complex and characterized by widely divergent reactions to similar forces, what, then, are the factors that raise or lower vulnerability, in the past as well as today? Building on insights from structural demographic theory (SDT), ongoing research by the Seshat team and other colleagues [29,31–35] demonstrates that societal pressure rises with the combination of three central structural stressors: *popular immiseration*, namely declining well-being and living standards for the majority population; the *elite overproduction and conflict* that arises when a growing number of wealthy and powerful citizens along with aspirant elites vie for limited economic, political and social prestige positions; and *state fiscal distress and declining state function* as resources are constrained and frustration at poor conditions erodes state legitimacy and capacity. These pressures tend to build over time owing to rising population and unequal distribution of wealth and opportunity, which depresses living standards among large segments of the population and raises the stakes of intra-elite competition. Further, as state finances and authority weaken or are corrupted by these same forces, their coordination and conflict-resolution mechanisms become increasingly ineffective, further undermining legitimacy [34].

Understanding how these pressures build and the ways that they increase social vulnerability necessarily involves a science of culture, as a primary issue at stake here is the ability of societies to engage in collective action at scale: to maintain governing systems and infrastructure, to support economic and cultural activities or empower political actions, and—most critically for the current paper—to navigate environmental threats and adapt to new realities. Indeed, the ability to scale-up collective action among increasingly large, diffuse and diverse assemblages of people is fundamental to maintaining societal functions and has been identified as critical in the evolution of increasingly large and complex social systems [36–39]. Our research using the SDT framework shows that, as the structural pressures identified above mount, societies can increasingly fragment and polarize as interest groups become frustrated, competitive, and hostile to each other and the prevailing government regime. Social cohesion erodes, catalysing unrest and animosity that often precipitates further frustration, polarization and conflict. In such an environment, it becomes increasingly likely that some ‘triggering event' will arise—a protest, an assassination, an economic downturn, a major ecological stressor or any number of other social disrupters. At the same time, the growing pressure makes it harder for the prevailing sociocultural systems to maintain resilience or effectively reorganize following a crisis; the society essentially lacks the requisite collective action capacity. As part of this, social inertia among groups with entrenched power, wealth, and social privilege can impede effective adaptation in responding to threats by trying to maintain the ‘status quo' even as conditions worsen. This can ultimately exacerbate the underlying pressures and hasten a significant disruptive—often violent—and potentially transformative adaptive outcome.

**Figure 3.**
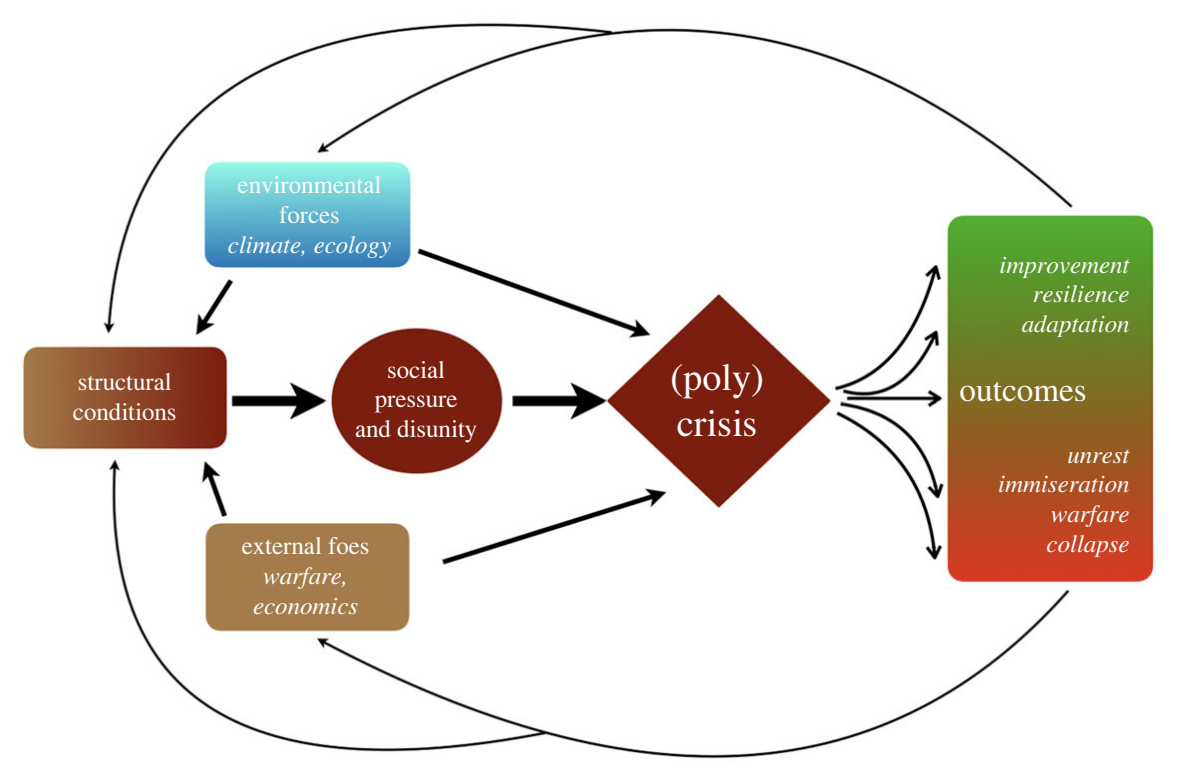
Flowchart illustrating idealized causal connections between different factors as discussed in the text.

SDT can, in our view, help explain why polycrises appear recurrently throughout history, as different elements of a society's structure feed back on each other in interaction with external forces, generating pressures that erode cohesion and cooperation among (and between) different structural elements within that society. For instance, an anomalous climate phase or sudden ecological shock might induce food shortages that propel popular immiseration and unrest, entrenched interests and intra-elite conflict might simultaneously work to thwart adaptive responses to changing environments or bristle as new sources of wealth and power arise. Additionally, the costs of responding to recurrent shocks or prolonged adverse conditions can drain state coffers, leaving the state unable to respond effectively to future disruptions. Even if some environmental forces are deemed truly exogenous causal influences triggering disruption, their impact can *only* be fully understood in the ways that they interact with, and are mediated by, prevailing social structures (figure 3).

Environmental anthropologists and political scientists have highlighted similar processes and offered important alternative framings to explain them. Notably, several scholars highlight that for non-elites (and elite aspirants), sufficiently severe crises can become ‘revelatory', exposing inequalities or flaws in the status quo and result in adaptive social change.^9^ The transformative potential of such crises might then be operationalized if a ‘state of exception' also prevails, whereby the sheer scale of disruption promotes a suspension of business-as-usual, undermining or eliminating the authority of previously established power-holders, facilitating change and adaptation. Here, we focus on SDT as a useful means to reconcile insights on multiple different scales that, in our view, compliment other approaches.

Put simply, a society's evolved (or evolving) structures make them more or less *vulnerable* to threats arising either endogenously through the workings of the system itself or exogenously as a consequence of environmental changes (human-driven or ‘natural'). In a context of high social pressure, an external stressor can exacerbate unrest and instability that was already developing. By the same token, the lower these pressures are when a threat rises, the more likely it is that the society will have the cohesion and resources to mitigate the threat's impact, maintain well-being, or the resilience to quickly rebuild critical infrastructure. While further research is underway to quantify empirically how strong these effects are, this relationship between structural pressure and societal vulnerability is well documented and appears to go a long way towards explaining the wide range of outcomes observed in past cases of societal crisis, climate shifts and ecological shocks.

## 4. Societal responses to social and environmental stress: three cases

It is useful to illustrate these points by reference to three cases from our CrisisDB sample—the Qing Dynasty in China, the Ottoman Empire and the Monte Albán settlement in Mexico's Oaxaca Valley—taken from distinct regions and time-periods as well as being representative of differing social scales, levels of complexity and societal structures, each responding in different ways to environmental stressors. These examples suggest an approach to diagnosing environment–society relationships that accounts for the different scales and scopes at which relevant forces operate, and in which tracking how structural conditions change over time is deemed critical for revealing the pressures and behaviours that emerge as these various forces interact.

**Figure 4.**
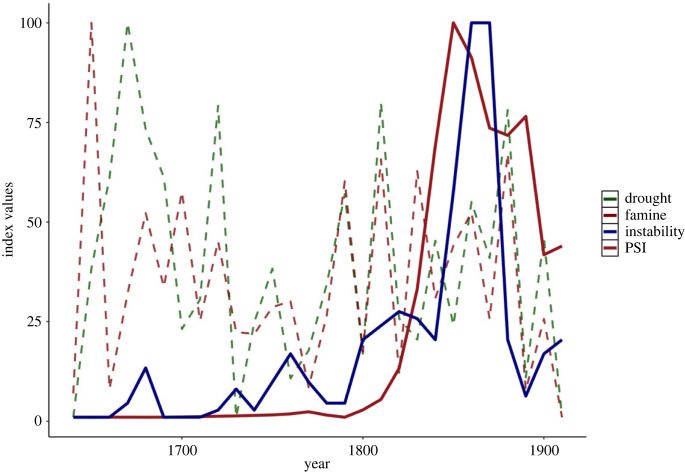
Ecological shock and social unrest in Qing China, 1640–1911 CE. Drought and famine record the number of sites in each year that experienced these shocks; instability is the number of years the Qing engaged in an internal war (civil war, violent uprising) per decade; and PSI is a composite measure of political–social instability, measured as the combined pressure from popular immiseration, elite overproduction, and state insecurity. Adapted from [34]. (Online version in colour.)

A recent analysis of the Qing, the last Chinese imperial dynasty, reveals clearly how social pressures mediate the impact of environmental forces [34]. The Qing were a large, economically productive, long-lived regime whose rule stretched from the end of the Ming Dynasty in 1644 until the Republican Revolution in 1911 CE. At the beginning of Qing rule, China was still experiencing a period of relatively cool temperatures and an unstable environment as part of the so-called Little Ice Age (figure 2). While there were several serious ecological threats in the first half of Qing rule, these did not lead to significant turmoil (figure 4). The Qing successfully maintained key state functions and mitigated impacts from ecological shocks through the eighteenth century by deploying state-run granaries and other measures to restore productivity and distribute resources to those needing them [41]. In the second half of the dynasty, however, social pressures rose sharply (figure 4) as overpopulation diminished available productive land for farmers, while the tightly regulated bureaucratic examination system kept a large number of elite aspirants frustrated at their lack of advancement; indeed, the Taiping rebellion (1850–1864 CE), often described as the bloodiest civil war in human history, was led by Hong Xiuquan, who failed the entrance exams for a top-ranking administration position five times along with many other frustrated elite aspirants. Making matters worse, the granary system, which had been so important in mitigating the impact of ecological threats in previous decades, became brittle with lack of support and rampant corruption among officials. Together, these mounting and mutually reinforcing structural pressures undermined Qing cohesion and, so, resilience. The ecological stressors faced during the ninteenth century were not dissimilar to those confronted previously but produced a sharp spike in instability, hitting a state no longer at an apogee of cohesion and stability, but one already in the throes of mounting social unrest, divisive partisanship and exhausted state capacities. The interaction of these pressures, added to competition from external powers like Britain, resulted in swelling instability through the ninteenth century that ultimately toppled the Qing and brought an end to dynastic rule in China.

A second illustration of such dynamics comes from another large and powerful imperial state, the Ottoman Empire. Environmental conditions during the sixteenth century were generally unfavourable across the Empire's vast territory, with most areas experiencing relatively low temperatures as part of the Little Ice Age (figure 2) which, as elsewhere, were associated with recurrent drought, harvest failures, famines and invigorated disease spread. Indeed, a particularly severe drought in the 1590s CE has been deemed ‘the longest in the Eastern Mediterranean for the past six centuries and by far the worst in the [Ottoman] empire's history' [42, p. 141]. The period from roughly 1519 to 1610 CE was also one of major internal unrest. The Celâlî Rebellions were a series of insurrections led by local elites frustrated at declining economic productivity and increasing competition for prestigious spots within the Ottoman administration. Several wars against external foes were also being waged, all combining to create serious social disruption. As a recent study puts it, ‘positive feedback between famine, violence, population displacement, and infectious disease led to population losses of 50% or more in parts of the empire [particularly the Anatolian heartland] between the 1580s and 1630s' – a 16th century ‘polycrisis' [43, p. 373]. As noted above, a similar mix of stressors has been cited as driving societal collapse in numerous instances, yet the Ottomans weathered these multiple storms and remained a large, stable, complex and fairly high-functioning state for years.

In part, the displacement and high mortality of this period relieved some of the mounting pressures from a growing population and dwindling *per capita* resources. Another critical factor is that the Ottomans maintained an intricate network of irrigation and drinking water infrastructure alongside social systems that distributed resources to those in need [44]. As with granaries under the early Qing, these systems helped to stave off cascading immiseration and unrest among large segments of the population; unlike the Qing, these systems were sustained until environmental–ecological conditions improved. State capacity and social cohesion remained relatively high during this period, as indicated by the state's ability to supress the rebellions in Anatolia, despite the costs of responding to recurrent stressors over a prolonged period. However, this stability must be balanced against the high mortality and unrest in parts of Ottoman territory. The sixteenth century polycrisis took its toll, but without spiralling into the catastrophic outcomes experienced by other societies facing similar challenges. Overall, the Ottoman experience underscores the importance of tracking how multiple forces interact leading up to and through periods of high stress, as outcomes can diverge even within a single society owing to regional variation in climate, ecology, structural pressures, resource availability and channels for distribution and capacity for coordinated response.

**Figure 5.**
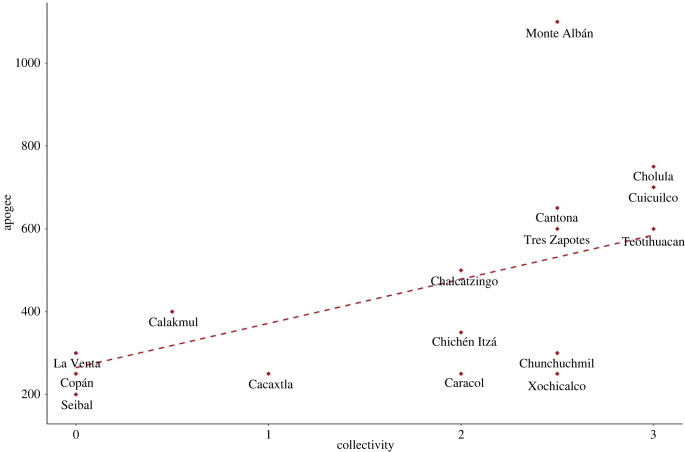
Correlation between the degree of collectivity evidenced in urban infrastructure and the length of apogee of site occupation in various Mesoamerican settlements during the pre-contact period (*n* = 26). Adapted from [45]. (Online version in colour.)

Our final case concerns the large Zapotec hilltop settlement of Monte Albán. Despite being situated in a fairly arid highland area in southern Mexico, subject to periodic drought, the site became the pre-eminent settlement in the Oaxaca Valley for more than a millennium from roughly 500 BCE. Settlement and governance patterns in Mesoamerica were varied, featuring highly centralized as well as more distributed power arrangements and socio-economic structures [45,46]. Early phases of Monte Albán have been noted for high degrees of collective action without highly personalized power, exemplified by public infrastructure and the urban layout: a lack of clear ‘palace' buildings and relatively flat differentials in the size of elite versus non-elite residences, many accessible temples and other civic buildings distributed throughout the site, small-scale water management and critical plaza spaces, which probably accommodated ritual, public and market activities. During the ninth century CE, Monte Albán experienced demographic decline, reduced control over its hinterland and diminished monumental building [47]. Some scholars view this as a major civilizational collapse, typically blamed on ecological disaster wrought by extended drought and inter-ethnic warfare, similar to the fate often ascribed to the Maya civilization and other Mesoamerican settlements around the same time (cf. [48]). More recently, however, this view has been questioned. Many aspects of Zapotec culture, including language, religious and economic practises, and certain domestic practises endured for centuries after, and indeed many remain today. Further, several other settlements in the region saw their numbers swell as Monte Albán's numbers declined, indicating resettlement rather than massive mortality. These areas, some in the driest sectors of the Oaxaca Valley, were probably subject to similar drought conditions as Monte Albán, belying the idea that these were ecologically forced migrations in any simple way. Rather, there are strong indications that Monte Alban declined owing to growing inequality and greater concentrations of power, which eroded well-being, fostered political polarization and diminished social cohesion. Notably, after around 800 CE elite residences became larger compared with the median house size, and the personalized iconography of rulers and elites became more prominent, signalling intensifying intra-elite competition. As Feinman and Nicholas state, many residents seem to have ‘voted with their feet' [47] to escape growing disparities in wealth and access to public spaces, perhaps exacerbated by the pressures of persistent drought. A similar pattern has been identified across Mesoamerica in the pre-contact period; sites with more collective forms of governance and public infrastructure generally exhibited greater sustainability in the context of changing ecological conditions (including droughts, floods, soil erosion and earthquakes) [45,49] (figure 5).

## 5. Critical directions for future research and policy implications: learning history's lessons

The above cases were selected to highlight how variable the impact of environmental stressors can be, not only across societies in different times and places, but even within a single society. It takes considerable cohesion and cooperation to develop critical infrastructure such as irrigation works for agricultural production, to maintain granaries to ensure food security, to manage quarantines or supply medical help during a disease outbreak, or to move resources to, and people from, areas affected by ecological shock. The more fractured and stressed that socio-cultural systems become, the less able a society is to enact such measures and the more devastating an external shock is likely to be. The historical record reveals that when these pressures are allowed to build over many years and external stressors are then added, the consequences are often severe, including significant unrest, civil violence and warfare and even regime collapse. Sometimes, however, unrest is minimal and violence largely constrained. Cases like the sixteenth-century Ottomans or experiences under the early Qing and Monte Albán highlight the importance of governance response to crisis and how a well-maintained infrastructure system can buttress against environmental catastrophe, along with effecting enough participation or ‘buy-in' from inhabitants to support these systems and their administration. More often, though, we find that power holders tend to ‘double-down' on prevailing systems and entrenched privilege during crises, often exacerbating tensions and unrest and thereby undermining attempts at adaptation and reform.^10^

On rare occasions the tension and dislodging of established systems during crises can facilitate meaningful institutional reform that boosts well-being for considerable numbers of people, increasing (or perhaps better, ‘reinvigorating') social resilience to future stressors. Sometimes this comes after a period of relatively minimal instability as with the Chartist period in Early Modern England (1838–1857 CE), which involved institutional reforms supported by a substantial segment of economic and political elites to increase wage and safety protections, especially for urban industrial workers. Alternatively, such reforms can result from extreme acts of disruption, massive mortality or in the aftermath of societal ‘collapse'. Examples of such ‘positive destruction' include the French Revolution, often cited as sparking the spread of modern, representative democratic governance throughout the western world; the legal abolition of slavery in the USA following a brutal civil war (1861–1856 CE); and how the devastation wrought by the bubonic plague in fourteenth and fifteenth centuries Europe spurred (for at least a period) higher wages for some labourers and perhaps created conditions for industrialization and economic growth (e.g. [52,53]). Ongoing research aims to uncover the critical forces that influence which path crises will take—from the societal structures that enhance or diminish cohesion to micro-level considerations underpinning behavioural responses from elite groups within a society.

As we illustrate here, SDT offers a compelling lens to understand how such pressures generate social stress and vulnerability, why they so often build up to critical points and ‘polycrises', and what consequences can be expected when facing stressors. Declining living standards and rampant inequality breed dissatisfaction among large segments of the population, while wealthy and politically powerful elites often engage in increasingly tense partisan conflict owing to their own frustration at growing competition for often scarce prestige positions. This elite conflict typically involves subverting social and political norms, as increasingly extreme positions and measures are taken to ‘stand out' in a crowded field—whether invoking uprisings against the prevailing rule, or forming fundamentalist cliques to siphon support from traditional or more moderate political movements. This again directly relates to social cohesion; as pressures rise and animosity between competing factions intensifies, the state loses legitimacy and capacity, making it increasingly difficult to accomplish major work that requires collective action at a significant scale.

The implications for contemporary societies are clear: as ecological shocks, economic disruptions, inequality and major conflicts are apparently increasing in frequency and intensity within our current polycrisis, the imperative to relieve the structural pressures that belie cohesion and collaborative action becomes all the more pressing. An important direction for future research will be to untangle these patterns with reference to a wide body of empirical evidence, a task currently underway as part of the CrisisDB programme. In particular, more work is needed to explain why some crises lead to positive adaptations that raise well-being and/or inclusivity for different populations while others generate more oppressive, exploitative systems or spiral into destabilizing conflict. Here, we have outlined a holistic framework that, we contend, holds great promise to explain these complex and seemingly intractable patterns by emphasizing interactions between structural pressures, environmental and other external stressors, and evolved socio-cultural norms and capacities.

For the lessons of past experiences to make a meaningful impact on contemporary challenges, further engagement with government officials, advocacy groups and other stakeholders is needed. Such efforts are already underway. Projects and research communities have been organized seeking to derive policy-relevant findings from surveys of how societies in the past fared when facing climate and especially ecological challenges, including the past4future group, the Ecological Futures project, future earth's IHOPE group, the Cascade Institute, the Planetary Limits Action Network, the Hot or Cool Institute, TMP Climate and many of the Past Global Changes (PAGES) working groups—to name a few involving a growing cross-disciplinary constellation of scholars seeking to share insights with government officials, corporate leaders and other advocates. However, much more work remains to address the host of unresolved questions about the complex relationship between environmental and societal dynamics: what are the full range of responses that past societies have taken when facing ‘polycrises'? Which were successful in mitigating violence and disruption to societal functions while generating future resilience and improving popular well-being? Which failed or even backfired, exacerbating tensions and propelling vulnerability? What interventions, reforms or behavioural shifts offer the best prospects of decreasing vulnerability while boosting resilience and adaptability within our current polycrisis, typified by more permanent and human-driven climate and ecological change than in past experiences?

What is clear is that no understanding of how environmental stressors have impacted societies in the past, let alone how they are likely to shape our collective futures, can proceed without seriously engaging with the way that social structures and cultural traits evolve over long timescales. For scholars, the challenge moving forward is to uncover ‘leverage points' that can help shape the experience of societies facing crises away from destabilization and violence and towards stability and even positive reform. For government officials and other stakeholders, it is imperative that new policies ‘listen to the science' which *must* include not only biophysical, economic and political sciences but also the growing sciences of culture and cultural evolution as well as insights from history and other humanities research. We believe that a comprehensive framework as we have outlined here, one that explicitly considers not only how different factors interact at different scales, but also one that is sensitive to how social structures change over long timescales, from decades to centuries, offers the best path forward in developing strategies, interventions and preparing to navigate the challenges we will continue to face as the present polycrisis unfolds.

## Data Availability

Data for the severity of outcomes are provided as a downloadable csv file from the Dryad Digital Repository: https://doi.org/10.5061/dryad.mkkwh715m [54]. This is part of the Climate Change Adaptation Needs a Science of Culture data portal from the Dryad Digital Repository: https://doi.org/10.5061/dryad.bnzs7h4h4 [55]. This includes the values for each variable along with description of sources consulted and brief descriptions explaining coding decisions in each case, as is typical of the Seshat coding procedure. These data are also are provided as part of the Seshat dataset, available from the project website: seshatdatabank.info/databrowser. The data are provided in the electronic supplementary material [56].

## EndNotes

^1^ For recent overviews of such work and discussion of the challenges and opportunities involved in tracing historical environment–society interactions, see [9–11].
^2^ This view is consistent with recent calls by many scholars of climate adaptation and resilience, who stress that what is often labelled a ‘disaster’ is just one particular *outcome* of an ecological–environmental stressor in a given time and place—a major flood, hurricane, earthquake, drought, etc. Such a stressor becomes disaster only when prevailing social, political, economic and other systems prove unable to mitigate the impacts or maintain key functioning [16–18]. Less attention, however, has been given to uncovering factors leading these systems towards resilience and adaptability or vulnerability.
^3^ While we lack the space here to offer a full quantitative analysis of these data, in this contribution we discuss some critical insights arising from analyses revealing the complex relationship between environmental forces and societal dynamics. Forthcoming work delves more deeply into the patterns revealed in these data.
^4^ The measure of severity here is additive (counting the number of ‘disruptive events’), which does not capture the severity or scale of any single event; how bloody a particular civil war was, for instance, regardless of how many other events co-occurred. Unfortunately, such direct measures of an event's impact are often unavailable in the historical record. Still, we can measure extent somewhat, as variables such as civil wars that lasted a century, population collapse and political fragmentation are by their nature more extreme events than, for instance, the assassination of a ruler or downward elite mobility; see the electronic supplementary material, for details.
^5^ For a recent survey of this literature, see [11].
^6^ The English Civil War (1642–1651) and ‘The Fronde’, a series of French civil wars in 1648–1655.
^7^ The major civil war preceding the English Civil War was the Wars of the Roses (1455–1485), which took place when France was experiencing a period of national unification that followed the Hundred Years War (1337–1453). The French Wars of Religion (1562–1598) was a much more severe crisis than the Fronde, and occurred when England was internally at peace. England was similarly internally at peace during the French Revolution (1789–1799).
^8^ For more detailed accounts of the structural pressures and divergent experiences of these countries, see [29,30].
^9^ For the interweaving of Jaqueline Solway's conception of the ‘revelatory crisis’ and Giorgio Agamben's conception of the ‘state of exception’ in this manner, see [40].
^10^ cf. discussions of maladaptive elite response to times of unrest, in the past and present [50,51].

## References

[1] Lawrence M, Janzwood S, Homer-Dixon T. 2022 What is a global polycrisis? Cascade Institute Discussion Paper; 2022–4:11. See https://cascade institute.org.

[2] Sinha A et al. 2019 Role of climate in the rise and fall of the Neo-Assyrian Empire. Sci. Adv. **5**, eaax6656. (10.1126/sciadv.aax6656) 31763452 PMC6853769

[3] Weiss H (ed) 2017 Megadrought and collapse: from early agriculture to Angkor. Oxford, UK: Oxford University Press.

[4] Haug GH, Günther D, Peterson LC, Sigman DM, Hughen KA, Aeschlimann B. 2003 Climate and the collapse of Maya civilization. Science **299**, 1731-1735. (10.1126/science.1080444) 12637744

[5] Diamond J. 2005 Collapse: how societies choose to succeed or fail. New York, NY: Viking Penguin.

[6] Ferguson N. 2021 Doom: the politics of catastrophe. New York, NY: Penguin.

[7] Harper K. 2017 The fate of Rome: climate, disease, and the end of an empire. Princeton, NJ: Princeton University Press.

[8] Manning SW, Kocik C, Lorentzen B, Sparks JP. 2023 Severe multi-year drought coincident with Hittite collapse around 1198–1196 BC. Nature **614**, 719-724. (10.1038/s41586-022-05693-y) 36755095 PMC9946833

[9] Degroot D et al. 2021 Towards a rigorous understanding of societal responses to climate change. Nature **591**, 539-550. (10.1038/s41586-021-03190-2) 33762769

[10] Cumming GS, Peterson GD. 2017 Unifying research on social–ecological resilience and collapse. Trends Ecol. Evol. **32**, 695-713. (10.1016/j.tree.2017.06.014) 28734593

[11] Hoyer D. Forthcoming Decline and fall, growth and spread, or resilience? Approaches to studying how and why societies change. J. World Hist. **34**. See https://osf.io/preprints/socarxiv/43rgx/.

[12] Costanza R, Graumlicj LJ, Steffen W. 2007 Sustainability or collapse?: an integrated history and future of people on earth, 518 p. New York, NY: MIT Press. (Dahlem Workshop on Integrated History and Future of People on Earth).

[13] Degroot D, Anchukaitis KJ, Tierney JE, Riede F, Manica A, Moesswilde E, Gauthier N. 2022 The history of climate and society: a review of the influence of climate change on the human past. Environ. Res. Lett. **17**, 103001. (10.1088/1748-9326/ac8faa)

[14] Preiser-Kapeller J. 2012 Complex historical dynamics of crisis: the case of Byzantium. In Crisis and transformation (eds S Jalkotzy, A Suppan), pp. 69-128. Verlag der Österreichischen Akademie der Wissenschaften (cited 1 July 2021). (Denkschriften der phil.-hist. Klasse; vol. 441). See https://austriaca.at?arp=0x002d9f55.

[15] Frankopan P. 2023 The earth transformed: an untold history, 736 pp. New York, NY: Knopf.

[16] Burton CG, Toquica M, Asad KMB, Musori M. 2022 Validation and development of composite indices for measuring vulnerability to earthquakes using a socio-economic perspective. Nat. Hazards **111**, 1301-1334. (10.1007/s11069-021-05095-9)

[17] Raju E, Boyd E, Otto F. 2022 Stop blaming the climate for disasters. Commun. Earth Environ. **3**, 1-2. (10.1038/s43247-021-00332-2)

[18] Kelman I. 2020 Disaster by choice: how our actions turn natural hazards into catastrophes. Oxford, UK: Oxford University Press.

[19] Ludlow F, Kostick C, Morris C. 2022 Climate, violence and ethnic conflict in the Ancient World. The Cambridge World History of Genocide. **1**.

[20] Lieberman V. 2003 Strange parallels: volume 2, mainland mirrors: Europe, Japan, China, south Asia, and the islands: Southeast Asia in global context, C. 800–1830. Vol. 2. Cambridge, UK: Cambridge University Press.

[21] Kottak CP. 1999 The new ecological anthropology. Amer. Anthropol. **101**, 23-35. (10.1525/aa.1999.101.1.23)

[22] Peregrine PN. 2020 Climate and social change at the start of the Late Antique Little Ice Age. The Holocene **30**, 1643-1648. (10.1177/0959683620941079)

[23] Anderson EN. 2019 The east Asian world-system: climate and dynastic change. Berlin, Germany: Springer. See https://link.springer.com/book/10.1007/978–3-030-16870-4.

[24] Gao C, Ludlow F, Matthews JA, Stine AR, Robock A, Pan Y, Breen R, Nolan B, Sigl M. 2021 Volcanic climate impacts can act as ultimate and proximate causes of Chinese dynastic collapse. Commun. Earth Environ. **2**, 1-11. (10.1038/s43247-020-00077-4)

[25] Hanhijärvi S, Tingley MP, Korhola A. 2013 Pairwise comparisons to reconstruct mean temperature in the Arctic Atlantic Region over the last 2,000 years. Clim. Dyn. **41**, 2039-2060. (10.1007/s00382-013-1701-4)

[26] Li B, Nychka DW, Ammann CM. 2010 The value of multiproxy reconstruction of past climate. J. Amer. Stat. Assoc. **105**, 883-895. (10.1198/jasa.2010.ap09379)

[27] Scheffer M, van Nes EH, Bird D, Bocinsky RK, Kohler TA. 2021 Loss of resilience preceded transformations of pre-Hispanic Pueblo societies. Proc. Natl Acad. Sci. USA **118**, e2024397118. 33911035 10.1073/pnas.2024397118 PMC8106319

[28] Sokoloff D. 2004 The Maunder minimum and the solar dynamo. Sol. Phys. **224**, 145-152. (10.1007/s11207-005-4176-6)

[29] Goldstone JA. 1991 Revolution and rebellion in the early modern world. Berkeley, CA: University of California Press.

[30] Turchin P, Nefedov S. 2009 Secular cycles. Princeton, NJ: Princeton University Press.

[31] Ortmans O, Mazzeo E, Meshcherina K, Korotayev A. 2017 Modeling social pressures toward political instability in the United Kingdom after 1960: a demographic structural analysis. Cliodynamics **8**, 113-158.

[32] Turchin P. 2003 Historical dynamics: why states rise and fall. Princeton, NJ: Princeton University Press.

[33] Turchin P. 2016 Ages of discord: a structural-demographic analysis of American history. Chaplin, CT: Beresta Books.

[34] Orlandi G, Hoyer D, Zhao H, Bennett JS, Benam M, Kohn K, Turchin P, Bashir MK. 2023 Structural-demographic analysis of the Qing Dynasty (1644–1912) collapse in China. PLOS ONE **18**, e0289748. (10.1371/journal.pone.0289748) 37595006 PMC10437944

[35] Blanton RE, Feinman GM, Kowalewski SA, Fargher LF. 2020 Moral collapse and state failure: a view from the past. Front. Polit. Sci. **2**, 8. (10.3389/fpos.2020.568704) (cited 18 October 2020).

[36] Blanton RE, Fargher L. 2016 How humans cooperate: confronting the challenge of collective action. Boulder, CO: University Press of Colorado.

[37] Carballo D, Roscoe P, Feinman G. 2014 Cooperation and collective action in the cultural evolution of complex societies. J. Archaeol. Method Theory **21**, 98-133. (10.1007/s10816-012-9147-2)

[38] Gavrilets S, Richerson PJ. 2017 Collective action and the evolution of social norm internalization. Proc. Natl Acad. Sci. USA **114**, 6068-6073. (10.1073/pnas.1703857114) 28533363 PMC5468620

[39] Turchin P. 2015 Ultrasociety: how 10,000 years of war made humans the greatest cooperators on earth. Chaplin, CT: Beresta Books.

[40] Dove MR. 2013 The anthropology of climate change: an historical reader. New York, NY: John Wiley & Sons.

[41] Will PE, Wong R, Wong RB. 2020 Nourish the people: the state civilian granary system in China, 1650–1850. Ann Arbor, MI: University of Michigan Press.

[42] White S. 2011 The climate of rebellion in the early modern Ottoman Empire, 377 p. Cambridge, UK: Cambridge University Press.

[43] Xoplaki E et al. 2018 Modelling climate and societal resilience in the Eastern Mediterranean in the last millennium. Hum. Ecol. **46**, 363-379. (10.1007/s10745-018-9995-9) PMC6015627 29997409

[44] Imber C. 2019 The Ottoman Empire, 1300–1650: the structure of power. London, UK: Macmillan Education.

[45] Feinman GM, Carballo DM. 2018 Collaborative and competitive strategies in the variability and resiliency of large-scale societies in Mesoamerica. Econ. Anthropol. **5**, 7-19. (10.1002/sea2.12098)

[46] Nicholas LM, Feinman GM. 2022 The foundation of Monte Albán, intensification, and growth: coactive processes and joint production. Front. Polit. Sci. **4**, 805047. (10.3389/fpos.2022.805047).

[47] Feinman G, Nicholas L. 2016 After Monte Albán in the Central Valleys of Oaxaca: a reassessment. In Beyond collapse: archaeological perspectives on resilience, revitalization, and transformation in complex societies (ed. R Faulseit), pp. 43-69, Carbondale, IL: Southern Illinois University Press.

[48] Winter M. 2008 Classic to postclassic in Four Oaxaca Regions: the Mazateca, the Chinantla, the Mixe Region, and the Southern Isthmus. In After Monte Albán: transformation and negotiation in Oaxaca, Mexico (ed. JP Blomster), pp. 393-426. Washington, DC: Dumbarton Oaks.

[49] Carballo DM, Feinman GM, López Corral A. 2022 Mesoamerican urbanism: indigenous institutions, infrastructure, and resilience. Urban Studies. (10.1177/00420980221105418)

[50] Rushkoff D. 2022 *Survival of the richest: escape fantasies of the tech billionaires.* New York, NY: WW Norton.

[51] Turchin P. 2023 End times: elites, counter-elites and the path of political disintegration. London, UK: Random House. See https://www.penguin.co.uk/books/447345/end-times-by-turchin-peter/9780141999296.

[52] Scheidel W. 2017 The great leveler: violence and the history of inequality from the stone age to the twenty-first century, 525 p. Princeton, NJ: Princeton University Press.

[53] Hoyer D. 2018 Pulling a little optimism out of a very grim account of global inequality. A review of the great leveler: violence and the history of inequality from the Stone Age to the twenty-first century by Walter Scheidel (Princeton University Press, 2017). Cliodynamics **9**, 130-142. (10.21237/C7clio9138349)

[54] Hoyer D et al. 2023 Data from: Navigating polycrisis: long-run socio-cultural factors shape response to changing climate. Dryad Digital Repository. (10.5061/dryad.mkkwh715m) PMC10505849 37718603

[55] Hoyer D et al. 2023 Data from: Navigating polycrisis: long-run socio-cultural factors shape response to changing climate. Dryad Digital Repository. (10.5061/dryad.bnzs7h4h4) PMC10505849 37718603

[56] Hoyer D et al. 2023 Navigating polycrisis: long-run socio-cultural factors shape response to changing climate. Figshare. (10.6084/m9.figshare.c.6793634) PMC10505849 37718603

